# Cognitive profile of patients with facioscapulohumeral muscular dystrophy

**DOI:** 10.1590/1980-57642021dn15-040015

**Published:** 2021

**Authors:** Vanessa Brzoskowski dos Santos, Jonas Alex Morales Saute, Laís Alves Jacinto-Scudeiro, Annelise Ayres, Rafaela Soares Rech, Alcyr Alves de Oliveira, Maira Rozenfeld Olchik

**Affiliations:** 1 Postgraduate Program in Rehabilitation Sciences, Universidade Federal de Ciências da Saúde de Porto Alegre – Porto Alegre, RS, Brazil.; 2 Department of Internal Medicine, Universidade Federal do Rio Grande do Sul – Porto Alegre, RS, Brazil.; 3 Postgraduate Program in Medicine: Medical Sciences, Universidade Federal do Rio Grande do Sul – Porto Alegre, RS, Brazil.; 4 Postgraduate Program in Health Sciences, Universidade Federal de Ciências da Saúde de Porto Alegre –Porto Alegre, RS, Brazil.; 5 Postgraduate Program in Epidemiology, Universidade Federal de Ciências da Saúde de Porto Alegre –Porto Alegre, RS, Brazil.; 6 Department of Surgery and Orthopedics, Universidade Federal do Rio Grande do Sul – Porto Alegre, RS, Brazil

**Keywords:** muscular dystrophies, neuromuscular diseases, cognition, quality of life, distrofias musculares, doenças neuromusculares, cognição, qualidade de vida

## Abstract

Although it is predominantly a muscular disease, impairments in the central nervous system in patients with facioscapulohumeral muscular dystrophy (FSHD) have been described in the literature.

**Objective:**

To describe the cognitive profile of patients with FSHD and to correlate the impairments found with clinical variables and quality of life.

**Methods:**

Cross-sectional and case–control study that evaluated FSHD patients using a series of cognitive assessments (Mini-Mental State Examination — MMSE, Montreal Cognitive Assessment — MoCA, verbal fluency with phonological restriction — FAS, categorical verbal fluency — FAS-cat, trail-making test — TMT, and Rey’s Verbal Auditory Learning Test); a neurological severity scale (Gardner–Medwin–Walton — GMWS); and a quality of life measurement tool (Medical Outcomes Study 36-Item Short-Form Health Survey).

**Results:**

Individuals with FSHD (13) and healthy controls (26) were paired by gender and age. Significant differences between case and control groups were found in MMSE, TMT A, and A7 (p≤0.05) and MOCA (p≤0.001) performances. A positive correlation was verified in long-term memory impairments and the age in which symptoms appear (r=-0.593, p=0.033). Regarding quality of life assessment, the emotional domain correlated to MEEM (r=0.657, p=0.015), TMT A (r=-0.601, p=0.030), and A7 (r=0.617, p=0.025) performances.

**Conclusions:**

Individuals with FSHD presented mild impairments in the performance of tasks that involve attention, planning, and long-term memory functions. Those impairments were associated neither with the disease duration nor with its neurological severity.

## RESUMO

Embora seja uma doença predominantemente muscular, alterações no sistema nervoso central em pacientes com Distrofia Facioescapuloumeral (FSHD) têm sido descritas na literatura.

**Objetivo:**

Caracterizar o perfil cognitivo de pacientes com FSHD e correlacionar as alterações encontradas com variáveis clínicas e qualidade de vida.

**Métodos:**

Estudo transversal, caso-controle que avaliou pacientes com FSHD por meio de uma série de avaliações cognitivas (Mini Exame do Estado Mental — MEEM; *Montreal Cognitive Assessment* — MoCA; fluência verbal com restrição fonológica — FAS; fluência verbal categórica — FAS-cat; *Trail Making Test* — TMT; e *Rey’s Verbal Auditory Learning Test* — RAVLT); uma escala de gravidade neurológica (*Gardner-Medwin-Walton* — GMWS); e um questionário (*Medical Outcomes Study 36-Item Short-Form Health Survey* — SF-36).

**Resultados:**

A amostra foi composta por 13 indivíduos com FSHD e 26 controles saudáveis, pareados por sexo e idade. A análise comparativa entre os grupos FSHD e controle mostrou diferenças significativas no desempenho cognitivo dos testes MEEM, TMT A e A7 (p≤0.05) e no MoCA (p≤0,001). Verificou-se uma correlação positiva com a idade de início dos sintomas e o prejuízo na memória de longo prazo (r=-0,593, p=0,033). Em relação à qualidade de vida, observou-se uma correlação entre o domínio de limitação emocional e os testes MEEM (r=0,657, p=0,015), TMT A (r=-0,601, p=0,030) e A7 (r=0,617, p=0,025).

**Conclusões:**

Pacientes com FSHD apresentaram alterações leves na realização de tarefas que envolvem as funções de atenção, planejamento e memória de longo prazo. Essas alterações não tiveram associação com o tempo de doença nem com sua gravidade neurológica.

## INTRODUCTION

Facioscapulohumeral muscular dystrophy (FSHD) is a hereditary neuromuscular disease, which is caused, most of times, by a contraction of the D4Z4 macrosatellite repeat in the subtelomeric region of chromosome 4 (4q35), which has a dominant autosomal heritage pattern.^1^,^2^,^3^ Its worldwide incidence revolves around 1/30,000 and the birth incidence is approximately 1/15,000, making FSHD as one of the most prevalent muscular dystrophy among adults.^4^ ^,^ ^5^ FSHD is predominantly characterized by progressive weakness and atrophy in facial muscles, in muscles that support the scapula, and in those muscles that cover the humerus; however, it also affects other body areas.^6^,^7^,^8^

Although it is generally a muscular disease, impairments in the central nervous system (CNS) in individuals with FSHD have been described in the literature. Some studies on cerebral nuclear magnetic resonance imaging (MRI) show gray matter loss in the left precentral cortex; in the anterior cingulate cortex; and in the frontal region, in addition to a high incidence of signal hyperintensities in the white matter.^9^ ^,^ ^10^

Apart from the evidence that suggests impairments in the CNS related to FSHD, few studies have evaluated the cognitive function of these individuals. Cognitive impairments in attention, memory, spatial perception, and concept formation have been reported in the literature.^11^ ^,^ ^12^ However, there is no standardized, objective, and detailed characterization of the cognition aspects and their relationship with FSHD; in addition, these data are unknown concerning the Brazilian population.

Thus, the objective of this study was to characterize the cognitive profile of patients with FSHD, and to correlate the impairments found with the clinical variables of the disease severity and quality of life.

## METHODS

### Study design

This was a cross-sectional, case–control, exploratory study.

### Subjects

Patients were recruited from an outpatient clinic, located at a hospital in Porto Alegre, Brazil, which is a reference in the treatment of individuals presenting neuromuscular diseases. The inclusion of participants took place from April to November 2019. Initially, the diagnostic criteria were reviewed on an electronic medical record. Patients with a clinical diagnosis of FSHD and aged 18 and above were screened. Subjects presenting other neurological or systemic conditions that could interfere with the test results were excluded. Healthy and unrelated individuals, matched for sex and age, were recruited from the community as a control group. Informed consent was obtained from all individuals prior to any study procedure. The project was approved by the institution’s Research Ethics Committee with the certificate of approval number 170552.

### Procedures

All study procedures were performed by the same assessor, who was trained for scales application and good clinical practices. Subjects were assessed in a private room located at the Hospital where the study was performed. The total duration of all procedures for each individual was approximately 50 min.

### Measures

#### Questionnaires

- Sociodemographic questionnaire: a structured questionnaire used to gather general patient data, such as age, gender, education, initial symptoms, and length of illness.
- *Medical Outcomes Study 36-Item Short-Form Health Survey* (SF-36): a quality of life assessment instrument, consisting of eight dimensions: physical function, role physical, bodily pain, general health, vitality, social function, role emotional, and mental health. Each dimension could be scored from 0 to 100 and higher scores were an indicator of better health.^13^

#### Motor scales

- *Gardner–Medwin–Walton* (GMWS): a clinical scale used to quantify the neurological severity of FSHD. The instrument is divided into 10° (0–9), increasing in severity.^14^

#### Cognitive tests

- Mini-Mental State Examination (MMSE): screening test translated and validated for the Brazilian population. The cutoff used for formal education is 28 points for more than 8 years; 26 points for 5 and 8 years; 25 points for 1 and 4 years; and 20 points for illiterate patients.^15^
- Montreal Cognitive Assessment (MoCA): screening test translated and validated for the Brazilian population. The cutoff point is 26 points; individuals with 12 or less years of education receive 1 extra point.^16^
- Verbal fluency with phonological restriction (FAS): validated test for the Brazilian population. The score is made by adding all the words spoken in the three letters; the score is affected by education and age.^17^
- Verbal categorical fluency (animals) (FAS-cat): validated test for the Brazilian population. A score of ≥9 named animals for subjects with up to 8 years of formal education and ≥13 named animals for those with over 9 years of formal education.^18^
- Trail-making test (TMT): the total score is given according to the time, in seconds, to complete each part of the test. The more time spent to completion, the worse the test performance.^19^
- Rey’s Auditory Verbal Learning Test (RAVLT): validated test for the Brazilian population. Scoring is assessed by age (20–59 and over 60) and gender (female and male).^20^

### Statistical analysis

Independent variables (e.g., age, education, age at disease onset, duration of illness, and GMWS) were presented as descriptive analysis (absolute and relative frequencies, as well as mean and standard deviation or median and interquartile range). The cognition tests of case and control groups were analyzed using Student’s *t-*test or Mann–Whitney U test taking into consideration whether the samples were normally distributed or not. Correlations between cognitive performance scores and independent variables were tested using the Spearman’s correlation test. Statistical significance was defined as p<0.05. The statistical software used was SPSS version 22.0.

## RESULTS

A total of 26 patients diagnosed with FSHD were initially screened. After review of exclusion criteria, 13 individuals were excluded because of the following reasons: 5 (19.23%) unsuccessful contact attempts; 4 (15.38%) not attending the scheduled evaluation visit; 3 (11.53%) refusals to participate in the study; and 1 (3.85%) individual under 18 years old. The final sample consisted of 13 subjects (distributed in 7 families) with FSHD. The control group was then composed of 26 healthy individuals. Sociodemographic data is presented in Table 1.

**Table 1.** Demographic data of the facioscapulohumeral muscular dystrophy and control groups.

	FSHD (n=13)	Controls (n=26)	p-value
Female	9 (69.23%)	18 (69.2%)	
Age	49.5 (13.2)	49.3 (12.7)	0.958
Education level	8.6 (4.1)	11.5 (2.7)	
Age of disease onset	42.7 (15.9)	–	
Disease duration	6.7 (5.9)	–	
GMWS	0–1 (2.6%)1–2 (5.1%)2–0 (0%)3–0 (0%)4–6 (15.4%)5–2 (5.1%)6–1 (2.6%)7–0 (0%)8–1 (2.6%)9–0 (0%)	-	

Data are expressed in the form of average years (standard deviation), except for the variables of sex and GMWS scores which are expressed in the form of frequency. FSHD: facioscapulohumeral muscular dystrophy; GMWS: Gardner–Medwin–Walton Scale.

Regarding cognitive assessment, impairments in the following tests were observed, according to the cutoff points: MoCA (76.92%), TMT A (100%), TMT B (69.23%), RAVLT (61.53%), short-term memory tasks (76.92%), and long-term memory tasks (61.53%) (Table 2). In individuals with FSHD, the quality of life related to health was affected in five from eight domains, most of them related to physical condition (Table 3).

**Table 2.** Descriptive analysis of cognition scores in the facioscapulohumeral muscular dystrophy group.

	Minimum	Maximum	FSHD (n=13)	Normal	Impairment
MMSE	23	30	26.76 (±2.45)	53.85% (7)	46.15% (6)
MoCA	18	29	23.00 (±3.51)	23.08% (3)	76.92% (10)
FAS	17	50	31.84 (±10.69)	84.62% (11)	15.38% (2)
FAS-cat	13	28	18.23 (±4.41)	100% (13)	0% (0)
TMT A	44	182	80 (58.50–138)	0% (0)	100% (13)
TMT B	57	291	123 (95.50–263)	30.77% (4)	69.23% (9)
RAVLT	20	61	34.53 (±12.09)	38.47% (5)	61.53% (8)
A6	4	15	6.76 (±3.05)	23.08% (3)	76.92% (10)
A7	3	15	6.30 (±3.42)	30.47% (5)	61.53% (8)

Data are expressed in the form of averages (standard deviation), except for the TMT A and TMT B test results which are expressed in the form of medians (interquartile range). FSHD: facioscapulohumeral muscular dystrophy; MMSE: Mini-Mental State Examination; MoCA: Montreal Cognitive Assessment; FAS: phonemic fluency test; FAS-cat: semantic fluency test (animals); TMT: trail-making test; RVALT: A6 and A7, Rey auditory verbal learning test.

**Table 3.** Descriptive analysis of quality of life scores in the facioscapulohumeral muscular dystrophy group.

SF-36	Minimum	Maximum	FSHD (n=13)
Physical function	0	85	30 (7.50–45)
Role – physical	0	100	0 (0–62.50)
Body pain	0	90	21 (15–46)
General health	15	95	27 (26–48.50)
Vitality	0	85	60 (25–65)
Social function	25	100	50 (31.25–75)
Role – emotional	0	100	33.50 (0–83.25)
Mental health	16	96	76 (42–89)

Data are expressed in the form of medians (interquartile range). FSHD: facioscapulohumeral muscular dystrophy; SF-36: Medical Outcomes Study 36-Item Short-Form Health Survey.

**Figure 1.**
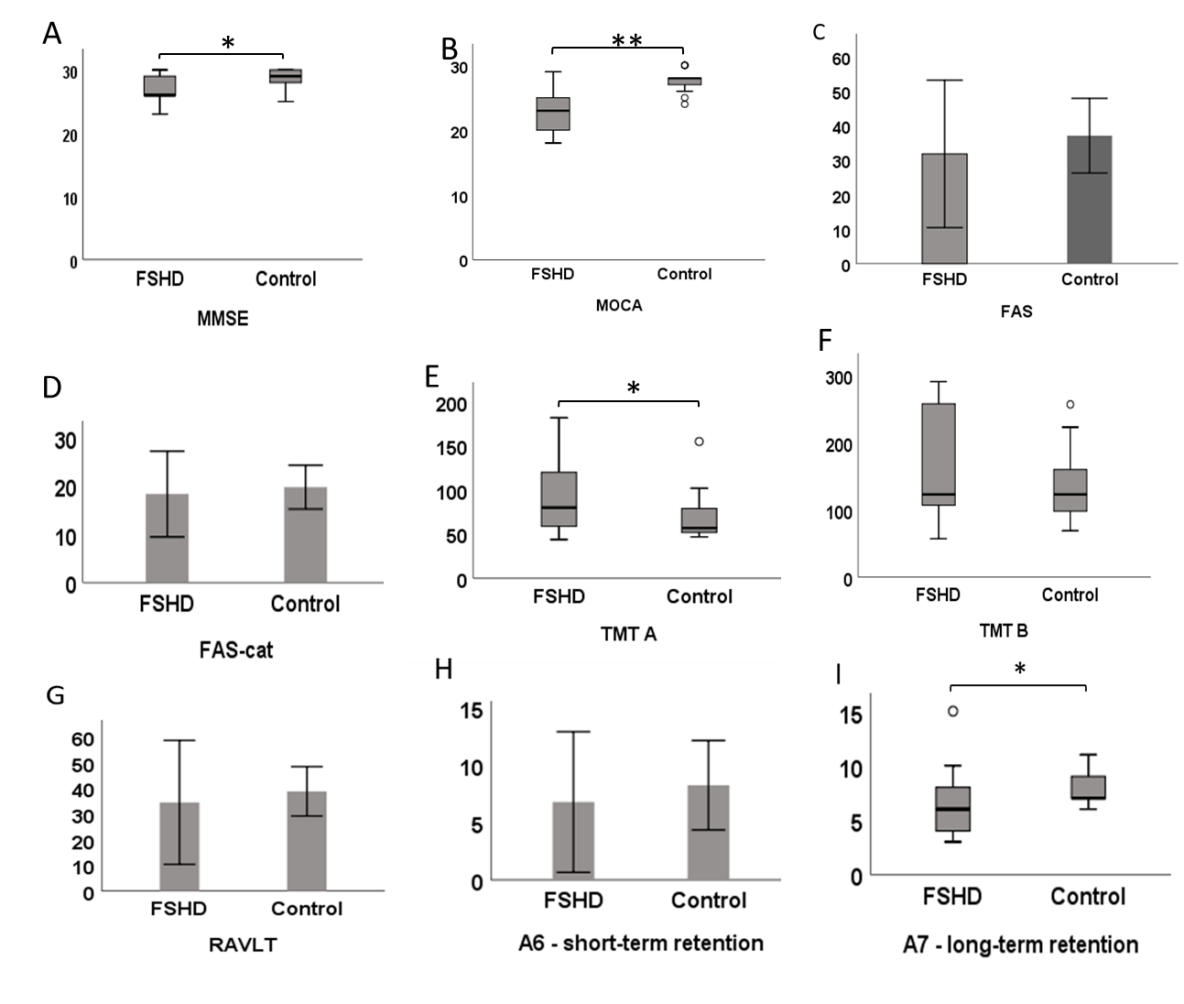
Comparison of cognitive test results between FSHD and control groups. Bars represent mean values and standard deviation. Box plots represent median values and interquartile ranges. *p<0.05 and **p<0.001. FSHD: facioscapulohumeral muscular dystrophy; MMSE: Mini-Mental State Examination; MoCA: Montreal Cognitive Assessment; FAS: phonemic fluency test; FAS-cat: semantic fluency test (animals); TMT: trail-making test; RVALT: A6 and A7, Rey auditory verbal learning test.

The comparative analysis between the FSHD and control groups showed significant differences in the cognitive performance of the MEEM, TMT A, A7 (p≤0.05), and MOCA (p≤0.001) tests as shown in Figure 1. Correlation tests were performed using raw scores of clinical variables and cognitive tests. The cognitive impairment correlation to the age of the symptoms beginning, time of the disease, and neurological severity is presented in Table 4.

**Table 4.** Correlations between cognitive tests and clinical variables.

	Initial symptoms		Lenght of illness		GMWS	
	p-value	r	p-value	r	p-value	r
MMSE	0.056	–	0.292	–	0.876	–
MOCA	0.173	–	0.422	–	0.578	–
TMT A	0.119	–	0.402	–	0.807	–
A7	0.033	-0.593	0.339	–	0.131	–

MMSE: Mini-Mental State Examination; MoCA: Montreal Cognitive Assessment; TMT: trail-making test; A7: long-term memory; GMWS: Gardner–Medwin–Walton Scale; Spearman’s correlation.

Correlation was found between cognitive tests and education, presenting higher scores related to the extension of the study: MMSE (r=0.677, p=0.011); MOCA (r=0.556, p=0.048); TMT A (r=-0.704, p=0.007); and A7 (r=0.644, p=0.013). There was also a correlation between age and the TMT A (r=0.578, p=0.039) and A7 (r=-0.628, p=0.022) tests, which were related to older patients.

Regarding quality of life, there was a correlation between the domain of emotional limitation and the MMSE tests (r=0.657, p=0.015), TMT A (r=-0.601, p=0.030), and A7 (r=0.617, p=0.025), showing that worse performances in cognitive tests were related to lower scores of emotional limitation domain.

## DISCUSSION

The present exploratory article describes a small sample of individuals with FSHD, whose decreased attention, planning, and memory tasks were not associated with the time or neurological severity of the disease. However, lower scores in cognition tests were correlated to worse results concerning the emotional limitation domain of SF-36.

Compared with previous studies,^11^ ^,^ ^12^ ^,^ ^21^ the subjects evaluated in our study presented a worse performance in cognition tests, which was related to the formal educational level. Our findings are in accordance with Zouvelou et al.,^12^ in which researchers identified memory and attention impairments, and with Sistiaga et al.,^11^ in which no significant correlation was found between cognitive test performance and neurological disease severity.

Due to the scarce literature about cognitive function in individuals with FSHD, it was opted to use a larger package of tests, once it was important to evaluate different aspects and functions. Besides that, two screening tests were used in order to ensure that a sensible instrument would be applied (MMSE and MoCA). The MoCA test seemed to be likely more sensible as a cognitive screening tool to be used in individuals with FSHD. More than half of the individuals with FSHD scored below the normal limits for MoCA when compared with MMSE tests. In addition, the discrepancy between MMSE and MOCA was also found in the compared groups analysis. Based on our study, it is possible to believe that MoCA can be a good screening tool for the continuous clinical evaluation of these patients. However, we emphasized the importance of a qualitative analysis of the test results followed by a referral to broader cognitive assessment in accordance with changes presented by the patient.

It is important to highlight that cognitive performance was not associated with the time or neurological severity of FSHD, which demonstrates that the impairment in this aspect does not seem to follow the progression of disabilities caused by muscular weakness. Cognitive impairments probably are manifested in different or independent times at the disease course. For this reason, continuous attention to clinical conditions is fundamental for the early detection of symptoms. Thus, aiming at quality of life and better management in the therapy, we suggested that the cognitive assessment in these patients should be carried out right after the diagnosis and applied continuously during the course of the disease.

It was also found a correlation between the domain of emotional limitation and cognitive tests showing that even if the cognitive impairments are mild, they seem to already impact the perceived quality of life of these patients. In this sample, it was observed that patients had lower scores in all domains related to physical and mental health. Those results are congruent to Padua et al.’s findings, who mentioned that individuals with FSHD present significantly lower scores in the physical domain, predominantly regarding pain, which is likely to be related to muscle deformities caused by the motor deficit.^22^

In addition, it was highlighted that the importance of future studies that can evaluate depressive symptoms and relate to cognitive impairments in individuals with FSHD. Considering that the previous literature indicates that individuals with depression have cognitive decline in tests that assess memory, attention, and executive function.^23^,^24^,^25^ In addition to the association with depression, the literature also describes that cognitive impairments in these patients may be related to the genotype. Sistiaga et al.^11^ identified two distinct cognitive profiles in FSHD depending on the molecular defect: patients with a fragment size >24 kb showed a relatively normal cognitive pattern and those with a fragment size <24 kb showed a significantly reduced intelligence quotient and difficulties with verbal function and visual-constructive tasks. When analyzing the FSH group, without getting separated by fragment size, there was a statistical difference in short-term memory when compared with the control group.

There is not a single primary outcome, since this is an exploratory study. For this reason and due to the scarce population of patients with the condition, sample size was not calculated, which is a limitation of the study. Therefore, we suggest that prospective observational studies should be carried out in the future, comprehending larger samples. This would allow identifying the appearance of cognitive impairments and assessing its progression, comparing it to the development of the usual motor disabilities caused by FSHD. Another important limitation of our study is the lack of homogeneity between groups in years of education. However, due to the absence of studies that address this topic based on the Brazilian population, we believe that, even with this limitation, our study contributes with important data on FSHD.

The mild impairment in attention, planning, and memory tasks performance presented by FSHD patients was not associated with neurological severity or time of the disease. However, results showed that worse performances in cognitive tests were related to lower scores of emotional limitation domains in quality of life assessments.
